# Perspectives in genetic counseling for spinal muscular atrophy in the new therapeutic era: early pre-symptomatic intervention and test in minors

**DOI:** 10.1038/s41431-019-0415-4

**Published:** 2019-05-03

**Authors:** Clara Serra-Juhe, Eduardo F. Tizzano

**Affiliations:** 1 Department of Clinical and Molecular Genetics Hospital Valle Hebron, Medicine Genetics Group VHIR, Barcelona, Spain; 2 CIBERER, Barcelona, Spain

**Keywords:** Preventive medicine, Motor neuron disease

## Abstract

Spinal muscular atrophy (SMA) is an autosomal-recessive neuromuscular disorder representing a continuous spectrum of muscular weakness ranging from compromised neonates to adults with minimal manifestations. Patients show homozygous absence or disease-causing variants of the SMN1 gene (−/− or 0/0) and in carriers only one copy is absent or mutated (1/0). Genetic diagnosis and counseling in SMA present several challenges, including the existence of carriers (2/0) that are undistinguishable of non-carriers (1/1) with current genetic testing methods and the report of patients (0/0) with very mild manifestations and even asymptomatic that are discovered when a full symptomatic case appears in the family. Younger asymptomatic siblings of symptomatic SMA patients are usually never tested until adolescence or adult life. However, following regulatory approval of the first tailored treatment for SMA, the prospects for care of these patients have changed. Early testing, including pre-symptomatic newborn screening and confirmation of diagnosis would change proactive measures and opportunities for therapy based in the actual landscape of new treatments. This review discusses the challenges and new perspectives of genetic counseling in SMA.

## Introduction

Spinal muscular atrophy [SMA] is an autosomal-recessive neuromuscular disorder characterized by degeneration and loss of alpha-motor neurons in the anterior horn of the spinal cord. SMA is considered one of the commonest causes of infant morbimortality with an estimated incidence of 1/6000–1/10,000 live births [1].

Clinically, SMA patients present in general with some degree of muscle weakness, areflexia, respiratory, and orthopedic problems and are classified into four main groups based on motor milestones achieved and age of onset (Table 1). In type-I SMA patients (Werdnig–Hoffmann form), the disease begins before 6 months and patients usually die before the age of 2 years [2, 3]. Type-II SMA patients (intermediate form) manifest the disease before 18 months of life and they are able to sit but are unable to walk unaided. Type-III SMA patients show a milder form of the disease (Kugelberg–Welander form) after 18 months of life; these patients are able to stand and walk autonomously but often become wheelchair-bound during youth or adulthood. Subgroups for types I–III are considered here (Table 1). Type-IV SMA patients are characterized by onset after the age of 30 years with milder manifestations of muscular weakness. Finally, a fifth class might be considered, including those with minimal clinical manifestations and therefore usually undetected unless a haploidentical sibling shows SMA phenotype (see below last section). Altogether SMA represents a continuous spectrum of phenotypes ranging from very compromised neonates and infants to adults with minimal manifestations [4] (Table 1).

**Table 1:** Spinal muscular atrophy classification considering motor milestones achieved and the wide spectrum of manifestations of the disease including asymptomatic cases that are haploidentical to their corresponding more affected siblings (considered as type-V form)

Main SMA type	Subclassification	Onset	Milestones achieved	Evolution/natural history	Prevalent SMN2 copies	Representative references
I	Ia (also referred as type 0)^a^	Prenatal	None	Death in weeks Contractures Cardiopathy	1	[46]
I	Ib	<3 months	Poor or none cephalic control	Feeding and respiratory problems Linear declination Death after second or third year of life	2	[2, 3]
I	Ic	>3 months	Cephalic control	Feeding and respiratory problems Plateau in first 2 years	3	[2, 3]
II	IIa	>6 months	Sitter	Scoliosis May lose sitting capability	3	[7, 25, 47]
II	IIb	Usually after 12 months	Sitter	Scoliosis May stand with support	3	[7, 25, 47]
III	IIIa	Between 18 and 36 months	Walker unaided	Scoliosis Earlier loss of walking	3	[15]
III	IIIb	>3 years	Walker unaided	Later loss of walking	3–4	[15, 25]
IV	None^b^	Second/third decade of life	Walker unaided	Most of life walking	3–5	[44, 48]
V	None	SMN1 absence with minimal manifestations or asymptomatic	All major milestones	Complete life walking	3–5	[24, 40, 42, 44]

*SMA* spinal muscular atrophy, *SMN1, 2* *Survival motor neuron 1, 2*

^a^Some authors reserve the term type Ia to describe patients who are very severe starting in a few days to differentiate the typical congenital form as type 0. In our Table, we consider type 0 or type Ia as those patients in the extreme category of severity with one SMN2 copy

^b^Some authors consider type IV as a type IIIb with adult onset

The *Survival motor neuron 1* gene (*SMN1*) has been identified as the SMA disease-determining gene [5] caused by the occurrence of homozygous absence by deletion or gene conversion events (90%), hybrid genes (5%), or subtle disease-causing variants (<5%) [6]. This gene is located in a complex genomic structure with 500-kb duplication at 5q13. As a consequence of this duplication, in the centromeric region of this locus, there is a highly homologous copy of *SMN1* and *SMN2*, which has been described as an SMA modifier [5, 7]. Both genes are quite identical with some divergences mainly localized within the 3’ end of these genes [8]. A C>T transition at position +6 of the exon 7 (c.840C>T, NM_000344.3; exons are numbered as in Bürglen et al. [8]) is the only difference in the coding region and is responsible for alternative splicing of this exon. As a consequence most of the *SMN2* transcripts lack exon 7 and the protein is incomplete and rapidly degraded. The broad range of phenotypic severity is modified mainly by the number of copies of this “backup” *SMN2* gene. However, the correlation is not absolute and other genetic or environmental factors may influence the final phenotype in a patient [7].

Advances into the genetics of SMA have identified therapeutic targets that are now under clinical investigation (www.clinicaltrials.gov). Moreover, the first tailored therapy for SMA has been approved in US and Europe transforming the prospects for care, trajectories of the disease, and evolving of the phenotype of these patients [9]. In this perspective review, we discuss how these advances may influence the genetic counseling approach and the decision-making process in SMA families.

## The new therapeutic landscape

**Fig. 1:**
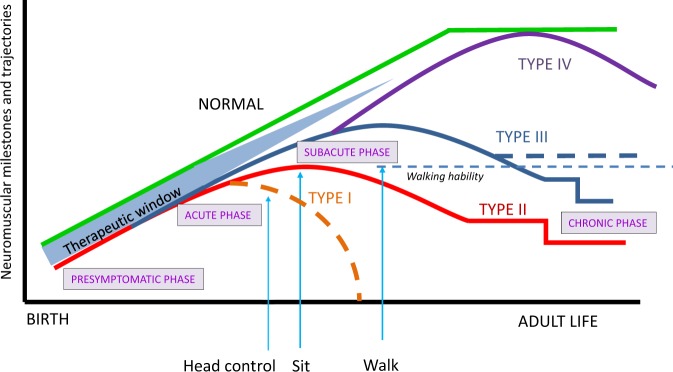
Neuromuscular milestones and trajectories from birth to adult life in spinal muscular atrophy (SMA) types. SMA patients present a pre-symptomatic phase, which is considered a therapeutic window to initiate the most effective intervention and treatment before substantial motor neuron loss occurs. Type-I disease has an acute phase with linear decline to death if no invasive respiratory intervention is made (dashed orange line). Type-II and -III SMA have a subacute onset in infancy and a late chronic more stable phase. Some type III patients maintain the walking ability for years (thick dashed blue line). Type-IV disease appears gradually during adult life. All SMA types can be treated early at pre-symptomatic stages according to the length of their respective and different therapeutic windows and is very likely that these patients will closely follow the green normal line. Treatment in already symptomatic patients may change trajectories with the slopes tending to reach the green normal line depending on each case (onset of disease, onset of treatment) and with new emerging phenotypes. Type-0 trajectories are not represented. Based on [4, 9, 12–16]

Clinical care and follow-up for SMA patients has advanced significantly over the past two decades. This is mainly due to improved proactive standard-of-care measures with the aim to minimize diagnostic delay for early intervention and optimize as much as possible quality of life and independent function [10, 11]. These supportive measures mainly include physiotherapy, respiratory care, and nutritional support, which do not affect the underlying biological progression of the disease. As a consequence, in type I patients motor function fails to improve and decline to become permanently ventilator dependent or a final fatal outcome [2, 3, 12]. In the less severe chronic forms, motor achievements may stabilize and later decline at specific ages being stable for long periods until the patients lose a fundamental motor milestone (i.e., autonomous sitting in very weak type-II patients or the walking capacity in type-III patients) [9, 13–15] (Fig. 1). The trajectory of the disease is now changing due to the success of novel therapies to improve motor function in these patients. The concept of SMA as an untreatable disease has now fundamentally evolved with the demonstration in clinical trials that nusinersen [Spinraza™] is effective and is the first approved drug for SMA treatment. Nusinersen is an antisense oligonucleotide that binds specifically a silencer sequence in intron 7 allowing inclusion of exon 7 in the mature mRNA from *SMN2* [4]. This mechanism increases the amount of full-length SMN, the protein that is diminished in patients with SMA. Clinical investigations demonstrate that SMA children can reach developmental milestones, which are never normally achieved in SMA [17], and in later onset patients nusinersen improves motor function [18]. Another advanced approach is gene therapy by *self-complementary* adeno-associated virus 9, which is still under clinical investigation but with very promising results in the first 15 treated type-I patients [19] and in other clinical trials ongoing. Several other medications are under study making SMA the rare genetic disease with most specific therapeutic options under clinical investigation including small molecules compounds that act as splicing modifiers, neuroprotectors, and myoactivators [www.clinicaltrials.gov]. As orphan drugs, their high cost and different refund policies of health insurance in different countries makes treatment not widely available. This implies a substantial commitment of different guilds such as health authorities and professionals, pharmaceutical industry, curators, and advocacy groups to guarantee equity and access to such treatments.

## Early intervention: toward pre-symptomatic testing and treatment

Preclinical data in SMA animal models [20] as well as results of clinical trials support the view that the earlier the treatment is initiated, the better the efficacy [17, 19]. This is observed in the ongoing Nurture trial in which early nusinersen treatment in pre-symptomatic infants prevents the onset of the SMA phenotype and allows for progressive achievements in motor function [21], NCT02386553, [www.clinical.trials.gov].

Pre-symptomatic individuals with SMA can be genetically identified by different situations. (1) Prenatal testing, requested by parents who have previously had an affected child or who are screened as carriers [22]. Much less common is the finding of an extended prenatal screening, even in the absence of any indication [16]; (2) Newborn screening, which is becoming available in some populations [23]; and (3) when one asymptomatic sibling is tested for *SMN1* to establish his/her carrier status but shows homozygous deletion as his/her affected sibling [24, 25]. If those subjects are treated pre-symptomatically, the latency period of the disease may be altered in different elements: age of symptom onset, weakness, and acquisition of motor milestones and evolution (Fig. 1). These modifications may range from remaining asymptomatic the rest of his/her life, delay the age of onset of weakness, or changing to an emerging phenotype [9].

The strategy that would have a greater impact regarding early detected patients is neonatal screening. In fact, with the impact of new therapies and treatments, a global change in healthcare policies should be done to take a crucial step forward in the approach of this disease. Strong arguments would support the addition of SMA in the newborn screening, considering the criteria proposed by Wilson and Jungner that are widely used to include or refuse a disease in the mentioned screening [26]. These criteria include 10 different items and all of them are virtually met by SMA (Table 2). The first two requisites deserve special attention: (1) the condition sought should be an important health problem, and (2) there should be an accepted treatment for patients with recognized disease.

**Table 2:** Wilson–Jungner criteria and references in support of their accomplishment in SMA

Criteria	Accomplishment	References
1. The condition sought should be an important health problem	Yes	[2, 3, 30, 31]
2. There should be an accepted treatment for patients with recognized disease	Yes	[17–19]
3. Facilities for diagnosis and treatment should be available	Yes	[10, 11]
4. There should be a recognizable latent or early symptomatic stage	Yes	[16, 27, 28]
5. There should be a suitable test or examination	Yes	[23, 49, 50]
6. The test should be acceptable to the population	Yes	[51, 52]
7. The natural history of the condition, including development from latent to declared disease, should be adequately understood	Yes	[2, 3, 12–15]
8. There should be an agreed policy on whom to treat as patients	Still debatable/ongoing	[9, 29]
9. The cost of case-finding should be economically balanced in relation to possible expenditure on medical care as a whole	Yes	[30, 31]
10. Case-finding should be a continuing process and not a “once and for all” project	Yes	[9, 53]

*SMA* spinal muscular atrophy

The annual incidence of SMA is approximately 1/6000–10,000 newborns, not far as the 1–5/10,000 newborns for phenylketonuria or 1/3500 for congenital hypothyroidism, two diseases worldwide included, and clearly higher than methylmalonic acidemia or medium-chain acyl-coenzyme A dehydrogenase deficiency, also included in some newborn screening programs. Regarding the treatment for patients, data proving the efficacy of newly developed drugs, as mentioned previously, would imply the compliance of this criterion.

A genetically confirmed neonatal asymptomatic case should be examined to look for features in the neuromuscular and respiratory phenotype (i.e., hypotonia, fasciculations, hypo-areflexia, paradoxical breathing) and other markers of disease [16, 27, 28]. The gradual implementation of universal neonatal screening would provide more information and experience to categorize each patient to observe the evolution together with the decision making by the family to receive treatment. The latency period to clinical onset and therefore the therapeutic window is larger in chronic type-II and -III forms than in acute type-I SMA and there is a longer evolution of the neuromuscular phenotype with less involvement of other systems or organs [9] (Fig. 1). Decision may be delayed to initiate treatment in some cases and proceed with a careful follow-up (see below) [29]. However, it also should be considered that improved care or proactive care of the positive asymptomatic cases may result in benefit for the clinical impact of the disease.

**Fig. 2:**
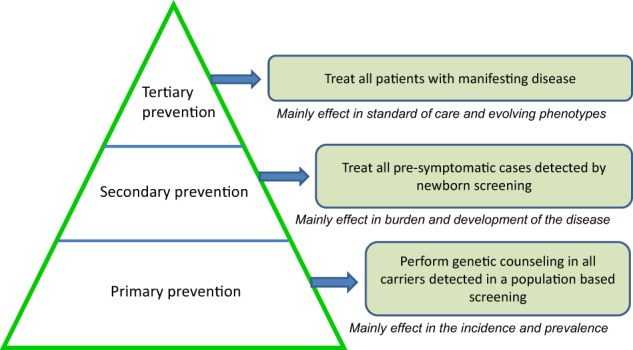
Epidemiologic evolution and prevention policies in spinal muscular atrophy (SMA). The actual situation of treatment of symptomatic cases when disease is already established (tertiary prevention, with a main effect in standard of care and evolving phenotypes) should change to implement newborn screening to treat patients before symptoms during the therapeutic window (secondary prevention, with a main effect in burden and development of disease). This would allow also detection of other carriers in the family. Public health policies should consider in the near future population carrier testing not only specific to SMA but several other autosomal-recessive conditions (primary prevention). These measures unavoidably will decrease the incidence and the prevalence of the disease in the future

At present, newborn screening is considered a reasonable initial approach for SMA prevention considering the moving from treat cases with manifesting disease (tertiary prevention) to treat disease in asymptomatic period (secondary prevention). However, it is envisaged that public health in the genomic scenario will result in the consolidation of carrier screening programs in the population (primary prevention) (Fig. 2). When carrier screening programs are implemented, couples have the opportunity to make a decision pre-conceptionally. In those cases in which both are carriers for the same disease, different options might be considered if the couple wants to avoid the disease in their offspring as prenatal testing, preimplantation genetic diagnosis, or gamete donation. These options cause a decrease of the incidence and prevalence of the disease with a substantial impact in the health system, considering the burden of the disease [30, 31] and the high cost of new treatments [32]. On the other hand, the availability of an effective treatment for the disease will probably increase the number of couples who may refuse the options mentioned above. Nevertheless, important ethical aspects emerged in these scenarios and genetic counseling should be provided including adequate and clear communication and psychological support to manage the expectations of the families.

**Fig. 3:**
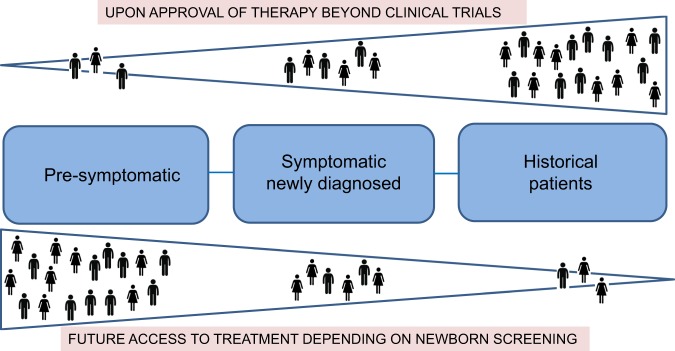
Changing scenario of access to treatment in spinal muscular atrophy. At present (upper triangle), the new treatments reach fewer pre-symptomatic babies, almost all newly symptomatic cases, and an important number of historical patients. The implementation of universal newborn screening (lower triangle) would modify the proportion and most of the patients will be treated at pre-symptomatic stages, fewer will be newly symptomatic (because of false negative in newborn screening, i.e., disease-causing point mutations), and the vast majority of the historical cases will be already under treatment

The availability of effective treatment options includes a strong debate of which pre-symptomatic SMA patients should be treated [9, 29]. At present, the only predictive marker that correlates in most of the cases with the different phenotypes is the number of *SMN2* copies [7]. More than 90% of the patients with two SMN2 copies will develop the most severe type-I disease starting in the newborn or infant period, whereas patients with four SMN2 copies will be virtually walkers and develop the milder forms even years later. Patients with three *SMN2* copies may develop whatever type of disease from type Ic (some cephalic control), type II (sitters), or type III (walkers) **(**Table 1**)**. No marker yet predicts if a patient with three *SMN2* copies will be a non-sitter, a sitter, or a walker [7]. Thus patients with three or four copies involve a difficult decision on when to start the treatment and proactive measures [7, 28, 29]. The actual scenario of treatment will modify the characteristic of the patients in the future, considering emerging phenotypes and updated classifications [9]. At present, most of the prevalent patients are historical with newly symptomatic diagnosed cases and a few pre-symptomatic detected. In the next future, the universal newborn screening is expected to transform this proportion and most of the cases would be treated in the pre-symptomatic period with a new evolving or even normal phenotype, fewer cases resulting from false negative in newborn screening may be detected late when manifesting the disease and none of very few historical will be naive of treatment (Fig. 3). Furthermore, the identification of the disease pre-symptomatically will also lead to the rapid identification of relatives at high risk of being carriers. This situation might be faced by genetic counselors to inform and manage family expectations and reproductive issues.

## Genetic counseling in SMA

### Carrier diagnosis

As an autosomal-recessive disease with a carrier frequency around 1/35–1/60 [1] in European populations, accurate detection of SMA carriers in a family is decisive for adequate genetic counseling. Accordingly, parents of a SMA case are not always carriers and a small proportion of them are considered as de novo or germinal/somatic mosaic cases influencing the final risk for a given couple [33]. Usually SMA carrier testing implies the performance of a quantitative method that detects one copy of *SMN1* in classical 1/0 carriers. A small proportion of carriers (~3–4%) have two *SMN1* copies in *cis* and none in the other allele (2/0 carriers). However, when an individual presents two *SMN1* copies, the current carrier diagnosis methods based on the *SMN1* dosage do not allow discrimination between 1/1 non-carriers and 2/0 carriers. In putative carriers within blood relatives, haplotype analysis located in the SMA locus is helpful to define these 2/0 carriers [33]. However, a more difficult genetic counseling situation involves the study of partners of SMA carriers (from the general population) with two *SMN1* copies where family tracking of haplotypes is not useful. Although the presence of some intragenic polymorphisms in *SMN1* is helpful to categorize a risk group of 2/0 carriers [34, 35], their absence in a person with two *SMN1* copies does not preclude 2/0 carrier status limiting the utility of this analysis to some populations [35]. Testing of the parents of a partner consultant to exclude the presence of a chromosome with one *SMN1* copy is usually confirmatory to interpret the consultant case as 1/1 non-carrier [33, 35] although the cost-effectiveness of this approach should be considered.

### Predictive biomarkers of evolution of disease

The number of *SMN2* copies that is detected in a patient is, to date, the most important known phenotypic modifier of the disease, since a higher number of SMN2 copies correlates with higher production of some properly spliced SMN mRNA and thus functional SMN protein levels leading to milder phenotypes. However, this correlation is not absolute and phenotypic discordances occur (Table 3). Patients with one or two *SMN2* copies usually develop type-I disease although a few patients present a milder phenotype [7]. The presence of a c.859G>C variant (exclusively to *SMN2*, NM_017411.3), which is a positive modifier, accounts for an important proportion of those milder cases [36, 37]. More recently, a G>A transition in intron 6 of *SMN2*, NC_000005.9:g.69372304A>G(Chr5,hg19), has also been described as a positive modifier [38]. On the other hand, patients with three *SMN2* copies may never walk or conversely may walk for several years. Patients with one copy (mostly type Ia or 0) or with four copies (mostly types IIIb and 4) are more consistent with the phenotype [7]. Thus *SMN2* copy number is a good (but not perfect) predictor of SMA type, albeit it is of no proven value as a marker of ongoing disease activity or response to treatment. For this purpose, other biomarkers such as neurofilaments are under study [39].

**Table 3:** Phenotypic discordances in SMA according to *SMN2* copies

Observed discordances	Situation	Possible explanation/cause	References
More severe phenotype than expected by the *SMN2* copy number	Type-I phenotype and 3 *SMN2* copies	Negative modifiers in *SMN2*	[7, 54]
Less severe phenotype than expected by the *SMN2* copy number	Type-II or -III phenotype and 2 *SMN2* copies	Positive modifiers in *SMN2*	[36–38]
Unrelated patients with 3 *SMN2* copies and different outcome (see also text)	All three main types, mostly sitters or walkers	Different transcriptional equivalence of *SMN2* copies (methylation,variants, partial deletions)	[7] and references therein
A haploidentical sibling less affected than his/her affected sibling or asymptomatic (with *SMN1* absence and the same *SMN2* copy number)	Dissimilar achieved motor milestones or minimal manifestations or asymptomatic	Genetic/genomic/epigenetic modifiers	[24, 40, 42, 44]

*SMA* spinal muscular atrophy

### Concordant and discordant siblings in SMA families

Asymptomatic younger siblings are usually not tested until they arrive to the reproductive age to request genetic counseling. Adult siblings of a SMA patient often seek for carrier testing and reproductive counseling. If the sibling under study presents one *SMN1* copy, this is confirmatory to be a carrier and testing can be offered to the partner. This apparently simple scenario of carrier testing and genetic counseling become complex when testing an asymptomatic sibling for *SMN1* copies and the result is 0 copies of *SMN1* as occurs with the affected person in the family [24, 40]. This rather unexpected finding opens the question of what will be the clinical evolution and outcome of this haploidentical sibling and, of course, if proactive measures or therapeutic options should be considered.

Most of these haploidentical sibs cases are detected in families with SMA milder forms in adulthood, and some of these particular siblings are considered as SMA with minimal manifestations or even asymptomatic (see Table 1). All these cases may be the result of different levels of SMN protein expression due to the presence of still unknown modifying genetic or environmental factors. Discordant phenotypes in SMA siblings have been reported prior to the identification of *SMN1* as causative of disease, and all cases appeared to have some degree of clinical manifestations [41]. After *SMN1* discovery, it was noted with surprise that even asymptomatic or unaffected siblings could be homozygously deleted for *SMN1* just as their symptomatic brothers or sisters. With a few exceptions, all these cases reported are haploidentical [24, 25, 40, 42 and references therein]. Thus the *SMN1* genotype and the number of copies of *SMN2* are the same within the siblings of a given family. Interestingly, in most of the families described in these studies, the less affected sibling tends to be a female. It has therefore been proposed that gender-related protective factors may influence the SMA phenotype. Indeed, it has been reported that unaffected *SMN1*-deleted females exhibited significantly higher expression of plastin 3 (*PLS3)* than their SMA-affected counterparts [42]. *PLS3* is considered a possible gender-specific SMA modifier whose protective effect may not be fully penetrant, thereby pointing toward an interaction with additional factors, such as neurocalcin [43]. However, there are no reported DNA variants in *PLS3* that can be applied as predictive of phenotype and discordance is not limited to male–female sibling pairs: it has also been reported in pairs of male siblings and in pairs of female siblings and in cases where females were more severely affected than males [24, 40, 44].

### Shall we perform early *SMN1* testing in minor asymptomatic siblings?

Genetic testing needs to be always considered from an ethical perspective given that genetic information is a special sensitive data in terms of confidentiality, privacy, and autonomy, including the right to know and the right not to know.

For these reasons, genetic testing in minors has been even more carefully addressed. While genetic testing to reach a diagnosis of a child clinically manifesting a genetic disease seems straightforward, the situation becomes complex in asymptomatic minors. Recommendations from different boards and institutions agree to only consider genetic testing in asymptomatic minors if the test result would modify their clinical management and, therefore, probably the outcome of the disease.

As stated by the *European Society of Human Genetics*, pre-symptomatic and predictive testing for conditions that become manifest in adulthood and that cannot be effectively treated or prevented, carrier testing should be discouraged until the person has the maturity and competence to comprehend its implications and, consequently, take an informed decision [45].

Regarding asymptomatic siblings of SMA patients, until now, genetic testing was mainly considered to explore their carrier status for their reproductive planning. Therefore, genetic counseling was offered when they reach reproductive age to ensure that the decision of undergoing a genetic test was taken by themselves with adequate information.

As mentioned before, in some cases, siblings of SMA patients were surprisingly found to be homozygous for a null allele when tested for reproductive risk assessment. Without an effective treatment, advancing this test in childhood would have implied a pre-symptomatic test for a disease that would become manifest later and that could not be effectively treated or prevented and thus not recommended by experts. Nevertheless, the scenario described above has substantially changed since effective treatments are being tested transforming the outcome and prognosis of the disease mainly with an early therapeutic intervention. Automatically, genetic testing for asymptomatic siblings of SMA patients would be reconsidered as a pre-symptomatic genetic testing for conditions with later (even adult) onset in which preventive actions can be initiated before manifestations, modifying the prognosis of the disease. In that case, genetic testing would be justified under the umbrella of the beneficence principle testing, as detecting a double null allele in an asymptomatic sibling of a SMA patient would inevitably activate the consideration of a close follow-up and a possible therapeutic intervention.

The described scenario not only compel us to reconsider genetic counseling approach and testing in apparently asymptomatic siblings but also urge the scientific community to include SMA in neonatal screening, considering the blooming of new therapeutic approaches.

In conclusion, the emergence of new and effective treatments boosts professionals to rethink the paradigm traditionally used to approach genetic diseases to ensure that innovation rapidly impacts on the patients’ quality of life.
